# A Patient on Long-Term Proton Pump Inhibitors Develops Sudden Seizures and Encephalopathy: An Unusual Presentation of Hypomagnesaemia

**DOI:** 10.1155/2012/632721

**Published:** 2012-11-18

**Authors:** Nirav Y. Gandhi, Walid K. Sharif, Sumeet Chadha, Jayadave Shakher

**Affiliations:** ^1^College of Medical and Dental Sciences, University of Birmingham, Birmingham B15 2TT, UK; ^2^Birmingham Heartlands Hospital, Heart of England Foundation Trust, Birmingham B9 5SS, UK

## Abstract

*Objective*. To present an unusual but known cause of hypomagnesaemia induced-hypocalcaemia in a chronic GORD patient with severe symptoms with a review of the current literature. *Methods*. Analysis of the clinical and laboratory findings of the patient and discussion of the multi-factorial nature of his disease and the underlying mechanisms. *Results*. Our patient described features of magnesium deficiency such as weakness, muscle twitches, and fits with clinical signs of hypocalcaemia: a carpal pedal spasm and paraesthesia. Preadmission blood results revealed low calcium and magnesium levels. He was admitted to ITU, when he presented with seizures and developed encephalopathy. The total vitamin D level was 52.4 nmol/L (>49.9). His U&Es and LFTs were within the normal range with the exception of potassium. He was on Omeprazole for his GORD. With omission of the PPI 1 day after admission and replacement therapy, his ion levels normalised. *Conclusion*. Hypomagnesaemia is often undiagnosed and is associated with multiple biochemical abnormalities. Treatment focus should be aimed at stopping the PPI and replacing the magnesium. Over use of PPIs is a problem in practice, with the FDA issuing a warning over long-term use. Continued monitoring and decision making on dose reduction/withdrawal is essential to avoid complications.

## 1. Introduction

In general, hypomagnesaemia is a usual finding in the hospital setting amongst patients, with reports suggesting incidences as high as 12% [1]. Most of these patients are in the ITU setting with gastrointestinal and renal losses as the main reasons of this derangement. Recently, drugs such as proton-pump inhibitors and diuretics are also known to cause or further contribute to a low magnesium level [2, 3].

PPIs such as omeprazole, which our patient was on, are potent inhibitors of gastric acid release from the parietal cells in the stomach. They inhibit a complex enzyme system: hydrogen-potassium adenosine triphosphate system [4]. In our patient, the PPI was used to treat his long-standing gastro-oesophageal reflux disease. Chronic PPI use can lead to the depletion of total magnesium levels, and some patients may acutely present with severe signs of hypomagnesaemia. Furthermore, a study showed that long-term PPI use nearly tripled the risk of developing bacterial gastroenteritis, whilst doubling the dose increased this risk to 5 times greater in comparison to the general population [5]. This can lead to aggravation of hypomagnesaemia in such patients.

Since 2007, the BNF include hypomagnesaemia as a side effect of PPIs [6]. It has been further suggested that these abnormalities cannot be corrected by replacing the total body magnesium via intravenous infusions, as seen in studies by Epstein et al. [7] and Agarwal et al. [8]. In both of these studies, magnesium replacement was not essential to achieving normal magnesium levels but merely stopping the PPI allowed electrolyte levels to return back to normality over time. Hence they speculate that PPIs have a role to play in some way by inhibiting gastrointestinal magnesium uptake.

We report a case of severe hypomagnesaemia-induced symptomatic hypocalcaemia due to Omeprazole precipitated by gastroenteritis. Hypomagnesaemia is usually associated with multiple biochemical abnormalities and can present with nonspecific symptoms such as weakness, tremors and muscle twitches. Magnesium level is not usually measured in a routine clinical setting, and high level of clinical acumen is required in patients on medications that are known to cause hypomagnesaemia.

We review the relevant literature and propose recommendations in relation to patient assessment prior to administering PPI therapy, in order to prevent PPI-induced electrolyte disturbances.

## 2. Case Report

A 67-year-old man of Caucasian descent presented to the Accident and Emergency Department at Birmingham Heartlands Hospital, Birmingham, on 25th of June 2012 with generalised lower abdominal pain, diarrhea, and vomiting. Prior to the patient calling the ambulance services, he had vomited 4 times in the past hour. This was on a background of a recent cruise trip abroad from which he returned on 22th of June 2012, whereby he describes eating a meat burger, which “didn't taste right.” 

The patient had no fever or any other indications of systemic disease. He complained of “weak” arms and legs and experienced bouts of paraesthesia and spasm of his fingers and toes, whilst in A&E. Subsequently, he deteriorated developing a carpopedal spasm and fits which required intubation and ITU care. The patient had no significant drug history except his long-term PPI.

On clinical examination, his abdomen was soft, nontender, there were no signs of any organomegaly, and bowel sounds were normal and present. There was no evidence of any cervical or axillary lymphadenopathy or goiter, and no cardiac murmurs. A respiratory examination showed clear lung fields. A neurological examination revealed no abnormalities. His blood results showed deranged electrolytes (see Table 1). Blood cultures and MC+S came back as negative. 

These findings were confirmed by another set of blood results (see Table 1). The total vitamin D level was normal at 52.4 nmol/L (>49.9). His urea and electrolytes were normal with sodium of 146 mmol/L, creatinine of 73 mL/minute, and LFTs in the normal range. However, his potassium level was low at 3.3 mmol/L.

On the 26th of June 2012, his PPI was stopped and the patient treated for hypomagnesaemia and hypocalcemic with intravenous replacement. Subsequently, he was prescribed oral calcium and vitamin D with ranitidine as a replacement for his PPI. He was discharged on the 6th of July 2012, when all his electrolyte levels had returned back to normal. His posttreatment PTH level of 12 pmol/L (1.6–7.2) suggests secondary hyperparathyroidism due to hypocalcaemia.

Table 1 lists the calcium, magnesium, potassium, and phosphate levels following his admission on the 25th of June 2012.

From Table 1, it is evident that the patient had consistently low level of magnesium associated with low calcium levels both of which returned to normal after discontinuation of omeprazole and intravenous infusions of both magnesium and calcium.

## 3. Discussion

Magnesium homeostasis is vital for many intracellular processes. It is tightly regulated by a dynamic relationship between gastrointestinal absorption, bone reservoir exchange, loop of Henle magnesium renal absorption, and renal excretion [9]. Since 2006, several studies have reported on the effects of long-term PPI use on reduction of gastrointestinal magnesium absorption.

It was estimated in 2008 that 113 million PPIs were prescribed in the United States alone [10], which led to the FDA's concern in 2011 on the consequences of prolonged PPI use and associated severe hypomagnesemia [11]. Tamura et al.'s large data mining study on FDA's Adverse Event Reporting System specified that Omeprazole was the highest ranked reported PPI (compared to other PPIs) to cause hypomagnesemia [12]. 

There are conflicting reports regarding the duration of PPI use to cause hypomagnesemia. Hoorn et al. stressed that a minimum of 1 year of continuous use would yield to reduction of magnesium gastrointestinal absorption [13]. However, Hess et al. [10] recent systematic review concluded that the onset of PPI-induced hypomagnesemia remains unspecific as the included studies ranged from 2 weeks to 13 years of use. In addition, they calculated an estimated median of 5.5 years of PPI use to induce hypomagnesemia. The above fits in with our patient profile, who was on omeprazole for at least 5 years. Moreover, it is important to note that most patients on long-term PPIs will not develop symptomatic hypomagnesemia [14] unless accompanied by precipitating factors such as gastroenteritis, diuretics, and chronic comorbidities. 

The biochemical mechanisms by which magnesium gastrointestinal absorption occurs can be divided into passive and active transport. The biochemical pathway of PPI and hypomagnesemia is yet to be confirmed; however, according to two studies [15, 16], omeprazole interferes with active intracellular transport by blocking H^+^/K^+^ATPase activity. The main reason behind this mechanism was suggested by Schlingmann et al. [17], where PPI-induced gastrointestinal pH changes will alter active transport.

It is important to appreciate the three-way relationship between magnesium, parathyroid hormone, and calcium. Epstein et al. [7] were the first to highlight the effect of hypomagnesemia on lowering PTH or to cause secondary hypocalcemia despite normal vitamin D levels. According to Freitag et al. [18], hypomagnesemia may suppress G-protein activation and cAMP production thus causing PTH resistance to calcium. Correction of magnesium levels leads to a rise in PTH levels, consequently increasing serum calcium. According to Mackay and Bladon [6], there is significant evidence to show that severe PPI-induced hypomagnesaemia was associated with hypocalcaemia in 64 % of cases.

Magnesium deficiency is frequently associated with hypokalaemia, which is refractory to potassium replacement. It has been shown that magnesium deficiency will cause a reduction in inhibitory effects on renal ATP-dependent ROMK channels, increasing basal potassium excretion [19]. 

## 4. Conclusion

Magnesium levels are maintained by equilibrium of both gastrointestinal absorption and renal tubular excretion. PPI use is a problem in practice and needs to be addressed, as long-term use is associated with hypomagnesemic hypoparathyroidism leading to secondary hypocalcemia. Therefore, continual monitoring and decision making on whether to reduce the dose/withdraw the PPI is essential to avoid complications. 

Hypomagnesaemia is often undiagnosed and is associated with multiple biochemical abnormalities. In summary, the management of PPI-induced symptomatic hypomagnesemia is to withdraw the PPI and initial correction with an intravenous magnesium infusion, in turn correcting both the calcium and potassium levels. It is acceptable to stop supplementation once magnesium levels are restored [20]. 

## Figures and Tables

**Table 1:** 

	Corrected calcium	Magnesium	Potassium
	(2.20–2.60 mmol/L)	(0.70–1.00 mmol/L)	(3.5–5.3 mmol/L)
Sample received	Value	Value	Value
25/06/12 18:09	1.85⇓	—	3.3⇓
26/06/12 07:09	1.71⇓	<0.27⇓	2.9⇓
26/06/12 10:17	1.80⇓	<0.27⇓	2.8⇓
PPI STOPPED			
26/06/12 16:29	2.06⇓	1.56⇑	—
26/06/12 21:43	2.16⇓	1.42⇑	—
27/06/12 05:52	2.26⇔	0.97⇔	3.8⇓
27/06/12 18:13	2.20⇔	0.87⇔	3.5⇔
28/06/12 07:20	2.13⇓	1.39⇑	4.1⇔
29/06/12 11:47	2.19⇓	0.89⇔	3.8⇔
05/07/12 15:56	2.37⇔	0.84⇔	4.7⇔

## Abbreviations

ATP: Adenosine triphosphate
A&E: Accident and Emergency
BNF: British National Formulary
cAMP: Cyclic adenosine monophosphate
FDA: Food and Drug Administration
GORD: Gastro-oesophageal reflux disease
ITU: Intensive care unit
LFTs: Liver function tests
PPI: Proton pump inhibitor
PTH: Parathyroid hormone
ROMK channels: Renal outer medullary potassium channels.
